# Exploring activities and behaviours potentially increases school-age children’s vulnerability to malaria infections in south-eastern Tanzania

**DOI:** 10.1186/s12936-023-04703-2

**Published:** 2023-10-03

**Authors:** Fadhila Kihwele, Tegemeo Gavana, Christina Makungu, Hajirani M. Msuya, Yeromin P. Mlacha, Nicodem James Govella, Prosper Pius Chaki, Bruno Fokas Sunguya

**Affiliations:** 1 Environmental Health and Ecological Sciences Department, #5 Ifakara Street, Plot 463 Mikocheni, P.O. Box 78373, Dar Es Salaam, United Republic of Tanzania; 2 Muhimbili University of Health and Allied Sciences, P.O. Box 65001, Dar es Salaam, United Republic of Tanzania; 3 The Pan African Mosquito Control Association (PAMCA), KEMRI Headquarters, Mbagathi Road Nairobi, Nairobi, 54840-00200 Kenya; 4 School of Life Sciences and Bioengineering (LISBE), Nelson Mandela African Institution of Science and Technology, P.O. BOX 447, Tengeru, Arusha, United Republic of Tanzania

**Keywords:** Malaria, Disease reservoir, School-age children, Focus Group Discussion, Human behavior, Outdoor malaria transmission

## Abstract

**Background:**

Strengthening malaria control activities in Tanzania has dramatically declined human malaria infections. However, there is an increasing epidemiological shift in the burden on school-age children. The underlying causes for such an epidemiological shift remain unknown in this context. This study explored activities and behaviours that could increase the vulnerability of school-age children to transmission risk to provide insight into protection gap with existing interventions and opportunities for supplementary interventions.

**Methods:**

This cross-sectional study conducted twenty-four focus group discussions (FGDs) in three districts of Rufiji, Kibiti and Kilwa in south-eastern Tanzania. Sixteen FGDs worked with school-age children (13 to 18 years) separating girls and boys and eight FGDs with their parents in mixed-gender groups. A total of 205 community members participated in FGDs across the study area. Of them, 72 participants were parents, while 133 were school-age children (65 boys and 68 girls).

**Results:**

Routine domestic activities such as fetching water, washing kitchen utensils, cooking, and recreational activities such as playing and watching television and studying were the reported activities that kept school-age children outdoors early evening to night hours (between 18:00 and 23:00). Likewise, the social and cultural events including initiation ceremonies and livelihood activities also kept this age group outdoors from late evening to early night and sometimes past midnight hours. Parents migrating to farms from December to June, leaving behind school-age children unsupervised affecting their net use behaviour plus spending more time outdoors at night, and the behaviour of children sprawling legs and hands while sleeping inside treated bed nets were identified as potential risks to infectious mosquito bites.

**Conclusion:**

The risky activities, behaviours, and social events mostly occurring outdoors might increase school-age children’s vulnerability to malaria infections. The findings provide preliminary insight on potential risk factors for persisting transmission. Further studies to quantify the risk behaviour and activities are recommended to establish the magnitude and anticipated impact on supplementary control strategies to control infection in school-age children.

## Background

Over the past two decades, Tanzania has achieved approximately 65% reduction of malaria cases across all age groups due to widespread of interventions including the use of effective anti-malarials, improved diagnosis, and malaria vector control [1]. Despite the impressive reduction, there is an increasingly epidemiological shift in the risk of infections toward school-age children across sub-Saharan Africa [2–10]. There is relatively highest proportion of asymptomatic malaria infection to school-age children, unlike previously when the risk was concentrated to under five of ages and pregnant women [2–11]. This raises significant concern especially now when most countries including Tanzania are aiming to reach the elimination of malaria by 2030 [12]. Unfortunately, underlying reasons for such epidemiological shift in risks remain unclear. Unless this is identified and addressed through a targeted approach, this group will continue to be a reservoir of malaria parasites [13], and sources for persisting malaria transmission, thus creating a risk for Tanzania to realize its goal of eliminating malaria by 2030 [12].

**Fig. 1:**
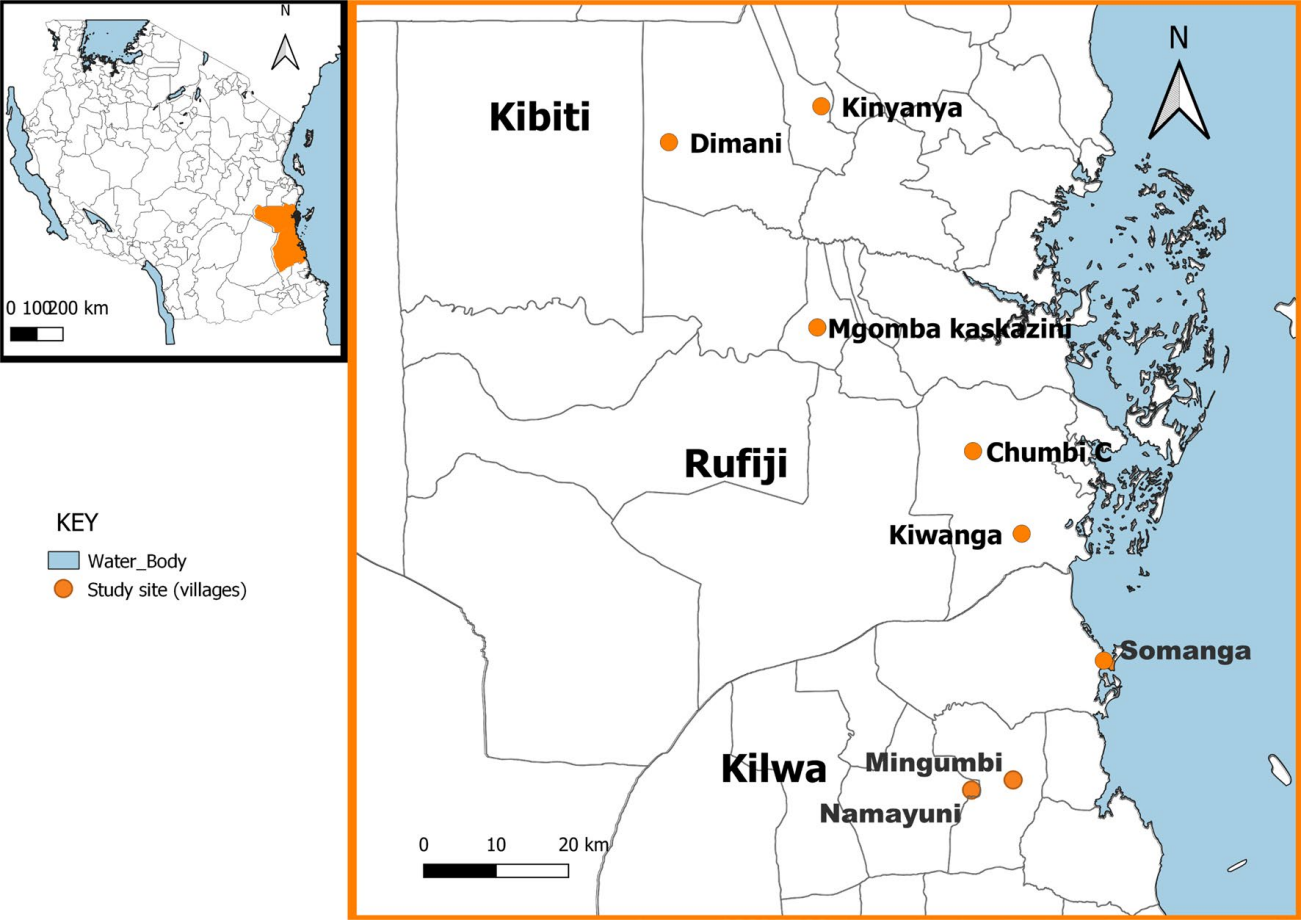
Map of the study area showing nine villages of Rufiji, Kilwa and Kibiti districts

The average malaria prevalence among school-age children stands at 21.6% nationally, this figure is estimated to be 14% higher than the under-five [14]. Several initiatives designed to interrupt and reduce malaria infection in this group including a school net distribution programme [12, 15–17]. The use of Insecticide-treated bed nets is the main malaria vector control in Tanzania. It is distributed to the community through different channels including targeted replacement campaign (TRC), reproductive and child health (RCH), and recently, distribution through primary schools, branded as School Net Programme (SNP). SNP is distributed annually to protect school-age children and as a keep-up strategy to maintain high access to ITNs to the population [15]. Even with this approach of which its implementation started since 2014, malaria transmission risk remains relatively higher to this age group. Pilot implementation research assessing the feasibility of chemoprevention for school children, known as intermitted preventive treatment (IPTs) demonstrated feasible in moderate and high malaria transmission settings in Tanzania. The National Malaria Control Programme (NMCP) of Tanzania is currently finalizing the guideline for the rollout of IPTs school in moderate and high malaria epidemiological strata. Despite these field-validated efficacious tools, a qualitative understanding of the drivers of the transmission that increases the risk of malaria exposure to this particular group needs to be elucidated to allow timely optimization of delivery or control strategies. This study explored activities and behaviours among school-age children that might be increasing their risk of exposure to malaria infections using a qualitative research approach only. It was aimed to set a foundation for further studies including quantification of relative contribution of each risk driver to inform the choice and prioritization of high-impact complimentary intervention by targeting the high-risk drivers (Fig. 1).

## Methods

### Study design and settings

A cross-sectional study using a qualitative approach was conducted in Rufiji, Kibiti and Kilwa districts in south-eastern Tanzania, where two phases of the China-UK-Tanzania project, “Application of Community-based Integrated Strategies to Reduce Malaria (ACOBISREM)” was implemented. Based on epidemiological stratification in Tanzania, the study areas fall within moderate to high transmission settings [18]. Malaria vectors responsible with malaria transmission in the settings include *Anopheles gambiae *sensu stricto (s.s.), *Anopheles arabiensis*, *Anopheles merus*, and *Anopheles funestus* s.s. [19]. Importantly, preliminary entomological investigation in this study area indicates some of such major malaria vector species start biting in early evening, time which coincides with the time when the majority of residents are likely to be outdoors and unprotected from front-line interventions, such as ITNs [20].

The Ifakara Health Institute (IHI) implemented phase one of the ACOBISREM project in high-burden areas of the Rufiji district, one of the districts of the Pwani Region in Tanzania, between 2015 and 2018 [5, 6, 21, 22]. The second phase was implemented from September 2019 to September 2021 in Rufiji, Kilwa and Kibiti districts to replicate and validate a scaling-up strategy. The intervention involved weekly community screening and treatment for malaria across the three districts. The community screening and treatment covered six villages per week and targeted all members of the selected villages. The screening was performed using the malaria rapid diagnostic test (RDT) and all asymptomatic malaria infections detected were treated using Dihydroartemisinin–piperaquine (DHA–PPQ). The intervention succeeded to reduced parasite prevalence by 66%. However, despite the significant reduction, the school-age children group carries the highest disease burden across the study area (Mlacha et al., pers. commun.).

### Theoretical framework

The ecological model was used as a conceptual framework to develop a rationale for conducting this study and design the topic guides. This model proposes to understand individual, social cultural and environmental factors in the setting to suggest a successful and cost-effective public health intervention [23]. Based on the ecological model the highest malaria infection among school-age children could be influenced by their individual, family and societal or community factors.

### Sampling and interviewees

School-age children aged between 5 and 18 years and adults with at least one school-age child who lived in the community for more than a year were recruited from three villages from each district exhibiting the highest asymptomatic malaria prevalence among school-age children. The highest malaria prevalence villages were selected based on data from the main study (ACOBISREM-project), which, had active weekly community screening for malaria across the three districts. The villages were purposively selected to capture diverse ecological conditions ranging from rural to peri-urban. Three focus group discussions (FGDs) with 8 to 12 participants were conducted in each village: one with parents (mixed group male and female) and two with school-age children (separating girls and boys). Separation aimed to ensure that they freely express their experiences and opinions. The research team purposively selected participants to ensure representation by age and gender in groups. Village leaders and teachers were involved in supporting the recruitment of participants and arrangements for data collection, all discussions were conducted either at village offices or at school in that particular village. Participants were asked to mention seasonal and routine activities conducted by school-age children outdoors in the early morning (05:00–07:00) and in the early evening (18:00) to the time they go to sleep as well as means of protection used by the age group when conducting the mentioned activities. Moreover, they were asked about net use and their perception of outdoor biting.

### Data collection

All FGDs were digitally recorded and audio records were repeatedly listened for consensus. FGDs were conducted in “*Kiswahili*” a common language in the area and nationally, and each discussion lasted approximately one hour. A total of twenty-four FGDs (nine each from Kilwa and Rufiji, and with six from Kibiti) were conducted. It was limited to six at Kibiti because of the saturation point [24, 25].

### Research team and reflexivity

The fieldwork activities for the current study were carried out by the first author FK and TG. All interviews were conducted by the first author, TG provided valuable assistance in recruiting study participants taking notes, obtaining informed consent, and contributing to the preliminary data analysis. Both FK and TG have been working as research scientists at the Ifakara Health Institute. Before the fieldwork, they received training in conducting interviews with children, ensuring confidentiality, and managing research data. FK’s background as a sociologist, along with her considerable research experience played a crucial role in skillfully establishing a good rapport with school children. This connection allowed her to gain their trust, facilitating the capture of valuable insights and experience from school children.

### Data analysis

Analysis followed a thematic framework approach by Richer and Spencer [26]. Audio records were saved in the computer and the external backup device and later transcribed and translated into English. Another person reviewed transcriptions for quality checks. Codes were generated thematically; transcriptions were imported to NVivo software version 12 for coding. Codes were deductively and inductively analyzed. Similarities and differences between themes as well as relations and patterns for developing categories were considered in the writing results [27].

## Results

### Socio-demographic characteristics of the study participants

A total of 205 community members participated in this study during data collection across the study area. Of them, 72 participants (45 male and 27 female) were parents, while 133 were school-age children (65 boys and 68 girls). The average age among school-age children and parents was 15.1 and 44.1 years, respectively. Parents reported agriculture as the main economic activity of all the mentioned occupations. In addition, 95.6% of all participants from the three districts can read and write (Table 1).

**Table 1:** Social demographic characteristics of FGD participants across the study area

Social demographic characteristics	Rufiji	Kilwa	Kibiti	Total
Number of FGDs conducted (n (%)				
School age children FGDs	6 (37.50)	6 (37.50)	4 (25.00)	16 (100.00)
Parents FGDs	3 (37.50)	3 (37.50)	2 (25,00)	8 (100.00)
Total	9 (37.50)	9 (37.50	6 (25.00)	24 (100.00)
Number of respondents participated n (%)				
School age children FGDs	48 (36.09)	42 (31.58)	43 (32.33)	133 (100.00)
Parents FGDs	29 (40.28)	25 (34.72)	18 (25.00)	72 (100.00)
Total	77 (37.56)	67 (32.68)	61 (29.76)	205 (100.00)
Age category, n (%)				
10–19	47 (35.61)	42 (31.82)	43 (32.58)	132 (100.00)
20–29	4 (28.57)	6 (42.86)	4 (28.57)	14 (100.00)
30–39	7 (50.00)	3 (21.43)	4 (28.57)	14 (100.00)
40–49	9 (47.37)	6 (31.58)	4 (21.05)	19 (100.00)
50–59	5 (411.67)	5 (41.67)	2 (16.67)	12 (100.00)
60 plus	5 (35.71)	5 (35.71)	4 (28.57)	14 (100.00)
Gender, n (%)				
Female	35 (36.84)	34 (35.79)	26 (27.37)	95 (100.00)
Male	42 (38.18)	33 (30)	35 (31.82)	110 (100.00)
Level of education, n (%)				
Secondary school	21 (45.65)	17 (36.96)	8 (17.30)	46 (100.00)
Primary school	47 (31.54)	49 (32.89)	53 (35.57)	149 (100.00)
Not attended school	9 (90.00)	1 (10.00)	0 (00.00)	10 (100.00)
Occupation, n (%)				
Pupils	18 (20.69)	26 (29.89)	43 (49.43)	87 (100.00)
Students	21 (45.65)	17 (36.96)	8 (17.39)	46 (100.00)
Agriculture	31 (53.45)	17 (29.31)	10 (17.24)	58 (100.00)
Livelihood/economic activities	7 (63.64)	4 (36.36)	0 (00.00)	11 (100.00)
Other	0 (00.00)	3 (100.00)	0 (00.00)	3 (100.00)

### Potential reasons for mosquito-bite exposures among school-aged children

Evidence gathered suggested seven potential main reasons for the increased risk of exposure to mosquito bites among the school-aged children in the study areas: (1) social activities; (2) cultural activities; (3) economic activities; (4) livelihood activities; (5) sleeping and waking up hours; (6) bed-net use behaviours; and (7) risk perception.

### Social activities

School-age children are mostly exposed to mosquito bites when they are outdoors from evening to bedtime hours. Social activities that would keep them outdoors at night were categorized into (i) routine social activities, (ii) studying (iii) social events and religious ceremonies.

i) Routine social activities
  Household chores and other evening activities performed by school-age children have been mentioned as social activities keeping this age group outdoors and sleeping late almost daily. Both parents and school-age children said routine evening activities such as cooking, bathing outdoors, cutting firewood, fetching water, washing utensils, watching television, socializing at home and spending time *kijiweni* (places where they gather for socialization), eating and playing outdoors at night are day to day activities that might subsidize school-age children exposure to a higher risk of contracting malaria. The routine activities done by the school-age children were gender-based as per Tanzanian social and cultural norms. Girls were said to be responsible for cooking and washing utensils while boys were spending time *kijiweni* while playing outside and cutting firewood were the shared activities.
  *“School-age children are playing outside in the late evening, and daily they are spending many hours watching television”* ***(Parent, Kilwa)***
  *“They are playing hiding games in long grasses where there are many mosquitoes, and parents cannot watch over the children all the times when they are playing”* ***(Parent, Rufiji)***
  Parents and school-age children highlighted that watching television programs, preferably series (local and international translated into the Swahili language) commonly known as *seasons*, is one of the exposure factors that may have contributed to high malaria prevalence in this age group. In peri-urban school-age children were watching *series and seasons* at home while in rural areas, children were watching programmes at the centres commonly called *kibandaumiza* in the Swahili language because the majority do not have television (TV) at home and others were gathering outside to watch TV from a person who has decided to bring their TV out so that anyone interested can watch freely. All parents in the study area said the school-age population, both boys and girls spend more time watching television, specifically the series and seasons after classes and during the weekend than other age groups in the communities. They paid 200 TSH to watch the programmes per day. These programmes start from 19:00 to 23:00 throughout the week. School-age children appeared familiar with the programmes, as they could identify the weekly schedule and broadcast time. Most parents across the study area said boys aged 15 years and older were returning home late because they were watching international football and local boxing matches in *kibandaumiza,* but this was a seasonal activity.
  *“Kulfi and Goodson’s series are broadcasted on Monday to Sunday from 19:00 to 21:00, while Mama Kimbo and the City series are from Monday to Friday from 19:00 to 23:00. But you can watch local movies on the Sinema Zetu channel anytime”* ***(School age child, Rufiji)***.
  *“Watching football is not a routine activity. Boys of 15 years and above are the ones going to watch football and boxing, coming back home at 24:00 to 04:00”* ***(School age child, Kilwa)***.
ii) Studying sessions (night academic sessions)
  Studying at night commonly known as preparation studies, is mandatory for national-examination year students (primary school-standard seven and secondary school form two and four students) and other students preparing for mid (in March and September) and end of the term (in June and December) examinations and monthly tests. Pupils and students reported going to classes from 19:00 to 22:30 for private study and returning home, but national examination candidates stayed in school camps under teachers’ supervision. School-age children confirmed not using preventive measures for mosquito bites when studying at night.
  *“Form one to form four students attend mandatory preparation studies session weekdays from 19:30 to 22:30, but we are not using prevention to mosquito bites because we are spending few hours in the class”* ***(School age child, Kilwa)***.
  *“At Chumbi primary school, there are examination preparation sessions from 19:00 to 22:00.”* ***(School-age child, Rufiji)***
iii) Social events and religious ceremonies
  Participants reported ceremonies and social events like wedding ceremonies happening during the dry season and immediately after harvesting in March to July as a potential risk factor that might increase the vulnerability of the age group to mosquito bites. During that time, people have money to host parties and the weather is favourable for outdoor parties. Most participants said parties included “v*igodoro*” (*Vigodoro is a kind of music played commonly at night after a social event like weddings and cultural ceremonies*). Music played included *singeli* and *bongo flavors*, which are mostly liked by youth, attracting school-age children to stay outdoors at night.
  *“I attend vigodoro parties during the weekends and normally return home at 05:00. We dance the whole night because they play all kinds of good music like bongo flavor, taarab and singeli. Sometimes famous musicians come to our villages.”* ***(School-age child, Kibiti)***.
  *“School-age children like Musics, specially vigodoro. They can spend a night or come home very late and if parents will not be stickily, even a seven years child can attend the party overnight*” ***(Parent, Kilwa)***.
  Islam is a common religion in the study area. Participants suggested *Dhikri* ceremonies as a factor exposing the school-age population to mosquito bites because it is conducted outdoors at night. Like other social ceremonies, Dhikri is commonly held after harvesting (March to July) when people have harvested and are financially well.
  *“Take an example of events like Dhikri, where people should sing and dance. These activities cannot be done while people are in bednet. By the way, we use bednets when people are in bed and not otherwise. They cannot dance with bednets”* ***(Parent, Kilwa)***.

### Cultural activities

Children’s initiation ceremonies involve a school-age population across the study area. It is usually conducted during the dry season and school holidays in June and December to avoid interference with school programmes. The practices are locally called *Jando* for boys and *unyago* for girls. This is when boys are circumcised, while Unyago is conducted for girls aged 12 to 15 when they get their first menstrual period. Most participants in peri-urban and rural areas said the modality of performing children’s initiations has changed because the majority are taking their boys to the hospital for circumcision instead of camping in the forest as it was in the past years. Contrary to that, participants in rural communities of Kilwa and Kibiti districts reported that *Jando* was still being conducted in the forest for 2 to 3 weeks. Boys slept in the forest with no protective measures against mosquito bites. Similarly, participants across the study area said *unyago* takes place at home. Still, initiation ceremonies for both girls and boys are held for 1 to 2 days outdoors, where a child gets gifts from parents and relatives as an incentive for the achievement.

*“In the past years, Jando was conducted in the forest to boys of 5 years and above but nowadays, circumcision takes place in the hospital and those who are still interested in traditionally circumcising their children are taking them to villages where they are still doing circumcision in the forest”* ***(Parent, Rufiji)***.

*“Jando is conducted in the forest and boys are staying there for 2 to 3 weeks”* ***(Parent, Kilwa)***

### Economic activities

The economic activities mentioned to be possible exposing school-age children to mosquito bites were mainly (i) agriculture which involved the migration of parents to the farm (ii) fishing.

i) Migration of parents to the farm
  Most parents migrate to farmlands from December to June for cultivation, harvesting activities and protecting crops from animals. Farms are far from their households, so migration to the farm is necessary to reduce daily go and return movements. Parents travel and reside in farmlands with the under-five children, leaving behind those studying with elder children or relatives without their parent’s care. Missing parents’ care can increase the risk of spending long hours of the evening and nights outdoors and not using bed nets or not using them correctly. Similarly, school-age respondents across the study area said their parent’s migration to Shamba gave them the freedom to watch television for long hours and spend more time outside. Several school-age children said younger children are at higher risk of sleeping without a bed net when parents are not at home because they cannot tuck bed nets properly. Therefore, there is a possibility of an increased risk of mosquito bites compared to when parents are at home.
  *“…when parents have moved to the farm, I get more time walking around the village and spend time at kijiweni because I am the in-charge in the household deciding the time to go back home at night”* ***(School age child, Kilwa)***.
  *“Parent’s care is unique and cannot be compared to whoever left with children when they migrated to the farm. A caretaker can order children to go to sleep, but parents would prepare a bed for their children and tuck in bed net for them before telling children to go to bed”* ***(Parent, Kilwa)***.
ii) Fishing activities
  Parents in Kilwa, specifically in Somanga village, said elder children who are not students are involved in fishing activities while those studying were doing the work during the weekends. Somanga is located in the peri-urban area near the Indian Ocean. Fishing is usually conducted at night, but sometimes they stay in the camps for a month or more.
  *“Some of 12 years children are not studying, have graduated standard seven or stopped studying, they are engaging themselves in fishing activities, which is normally done at night”* ***(Parent, Kilwa)***.

### Livelihood activities

Routine livelihood activities mentioned were selling food items like donuts, groundnuts, oranges, fish and boiled eggs conducted by school-age children in the late evening to night hours (18:00 to 24:00), exposing them to mosquito bites. School-age children did these activities to help their parents generate income.

*“Children of 11, 12 to 13 years are selling roasted maize, groundnuts, donates, cassava and oranges along the road”* ***(Parent, Rufiji)***.

*“During the weekend, they are helping parents generate family income by selling fish and boiled eggs”* ***(Parent, Kilwa)***

### Sleeping hours (time children are going to bed)

School-age children were going to bed late than other age groups in the community. They go to bed between 22:00 and 24:00. Watching television or attending social events are mostly everyday activities, keeping them awake too late at night. Adults above 18 years sleep between 21:00 and 22:00, while the under-five sleep immediately after dinner, between 19:00 and 20:00. There is a slight difference in hours they woke-up between school-age children and adults, while the under-five wake up late. The two age groups wake up between 04.00 and 05.00 when the school-age children are getting ready for school while those above 18 are getting ready for prayers (Muslims) or going to the farm, but most of the under-five wake up at 07:00. With no variation, all participants from peri-urban and rural said, all people in the community tend to sleep earlier during rainy season compared to dry season because of rain and cold weather.

*“Above 18 years are going to bed at 22:00 hours and the under-five normally sleep early at 20:00, but the school Aged 24:00. Watching television keeps them awake to late hours and if it is a match day, they can come home very early on the morning”* ***(Parent, Rufiji)***.

*“Adults above 18 are going to bed at 21:00 and the school age is sleeping around 24 hours because the school-age like to watch movies which normally ends at 23:30 while the under-five are sleeping at 20:00 hours.”* ***(Parent, Kilwa)***.

### Bed net use and sleeping behaviours among school-age children

Bed net remains the primary malaria prevention measure used across the study area, while only a few people use other measures like repellants, coils and wearing long sleeves clothes. Most school-age children in peri-urban and rural areas reported using bed nets distributed by school net programs in primary schools. Other sources of bed nets mentioned were antenatal and postnatal clinics and local shops. Bed net use behaviours are likely to increase school-age children’s vulnerability to mosquito bites if not properly used despite the reported high bed net use. Bed net use behaviours included (i) sleeping behaviours and *Ulalavi* (the tendency of sprawling legs and hands while sleeping), and (ii) laziness on tucking bed nets during the hot weather and lack of money to buy bed nets were reported as reasons not to use a bed net.

i) Sleeping behaviour and *ulalavi*
  Parents in this study area sleep with the under-five and reported giving them more attention than the school children. Both school-age children and parents said more than three children shared a bed because an extended family lifestyle is common across the study area. More importantly, *Ulalavi* was mentioned to be very common to younger school-age children than other age groups.
  *“….in our community, we call this behavior ulalavi, one sprawls legs and hands while sleeping, so if three children with this behavior sleep on one bed, they will touch the net. By doing so, mosquitoes will bite them and the bed net’s effectiveness will be low”* ***(Parent, Rufiji)***.
  *“Children start sleeping alone when reaching five years and parents’ care offered to the under-five is not the same as that given to school-age children. Very rarely, parents will check if the school-age children are using bed net or not because we believe they can take care of themselves”* ***(Parents, Kibiti)***.
  *“Sleeping behaviors of children are different to adults. Ulalavi makes them touch the bednets frequently and be at more risk of being bitten by mosquitoes than others.”* ***(Parent, Kibiti)***.
ii) Laziness to tuck in bed net
  School-age children were reported not to tuck bed nets because they are tired after many hours of playing. However, only a few parent participants said other school-age children are not interested in using bed nets despite their having and knowing the importance of using them. School-age children confirmed this and said the younger school children (5 to 10 years) are the ones who can fall asleep in any place, even outside the house, and if they don’t get immediate assistance, there is a high chance of sleeping without a bed net till morning.
  *“You can come back home late and at that time, children slept without bednet because of their laziness tucking the net”* ***(Parent, Rufiji)***.

### Risk perception on outdoor mosquito bites

Regardless of different levels of education, almost all participants across the study area believed mosquito bites in the evening hours before bed cannot transmit malaria because infectious mosquitoes bite only in the past midnight hours. Very few confirmed that being outdoors before bedtime is malaria risk behaviour and that people should use repellants that are available in local shops, but money has been mentioned as the central obstacle because the majority cannot afford to buy them. All participants perceived *ulalavi* (the tendency of sprawling legs and hands while sleeping) as a risk behaviour that could increase exposure of school-age children to infectious mosquito bites despite the routine bed net use.

*“We are not using protection when attending night studying sessions for a few hours from 20:00 to 22:00 because I know mosquitoes transmit malaria bites in the midnight hours and not before that time.”* ***(School-age child, Kilwa)***.

*“Mosquitoes that transmit malaria bites in past midnight only and at that time we are in bednets”* ***(School age child, Rufiji)***

## Discussion

This study intended to explore activities and behaviours of the school-age children that might contribute to their relatively higher malaria risk, by stressing more on net use behaviours and outdoor activities. The finding shows that routine and seasonal outdoor activities and risky bed net use behaviour are possibilities behind the persistently high prevalence of malaria parasites in the age group in the three districts of south-eastern Tanzania. The risk activities and drivers that might contribute to the current high prevalence included: routine night studying sessions, late night sleeping behaviour of school-age children compared to other age groups, migration of parents to farms, traditional initiation ceremonies, attending night social parties, and ulalavi (the tendency of sprawling legs and hands while sleeping).

Routine night studying sessions predispose school-age children to mosquito bites since they do not use protective measures. Unfortunately, entomological studies have reported a substantial proportion of biting mosquitoes before midnight (24:00) [28–31]. The under-five may be protected against such risk as they would be at home and under bed nets early. This difference in exposure time between school-age children and children under 5 years may be one of the fundamental reasons for the difference in the burden of malaria between children under-five and school-age children similar in another context [32]. In malaria-endemic areas, school-age children will remain vulnerable to malaria infections unless measures are taken to provide protective measures during night studying sessions [33]. This behaviour is a new phenomenon and currently increasing across the country as schools compete for better positions at the national level.

Attending seasonal social parties may be one of the main risk behaviours that might increase the age group’s vulnerability to malaria infection. In addition, the local music mostly liked by youth in the study and usually played from evening to past midnight hours attracts more school-age children than others. Currently, *vigodoro* is common and famous in Tanzania’s south and coastal zones while gaining popularity in other parts of the country. This practice, which has comprehensive geographical coverage and attracts a good number of school-age children, may continue to be a source of malaria infection in this age group if appropriate measures are not taken. Attending *vigodoro* has been a relatively new phenomenon and is favourite to school-age children over other age groups in the community. It has gained popularity in recent years in both rural and urban sites however, it is common in south and coastal zones and it became as part and parcel of their culture.

Traditional male initiation ceremonies can also be essential risky activities contributing to the malaria prevalence in the school-age group in rural sites in the study area. During the initiation time, a group of boys stayed in the forest for 2 to 3 weeks without using malaria preventive measures, despite awareness of the risk of malaria transmission at night. In Tanzania, male initiation ceremonies are common practice and it is an old phenomenon practiced in large parts of the country, especially the southern and coastal regions [34]. These are also areas with high malaria transmission and burden. Thus, it is likely that the practice puts a substantial number of school-age male children at risk of infections compared to other age groups. Although girls do not stay in the forest like boys, their initiation ceremonies are held at home, attracting relatives and friends in overnight celebrations. As a cultural aspect, these practices may not be possible to abolish, and thus continue to sustain transmissions unless measures are taken to protect the respective group.

From December to June, parents migrate to farmlands to protect crops from animals and conduct other agricultural activities. Parents migrated with the under-five and continued to be safe from mosquito bites. At the same time, the school-age children remained behind unsupervised, affecting proper net use. If the bed net is not used correctly, there is the possibility of being infected regardless of the daily net use [27]. Unfortunately, the farm’s seasonal migration pattern coincides with the high rainfall and increased breeding of mosquitoes in the study area [19] Parent’s migration to the farms is not a new behaviour among the agriculturalist in the study area and these findings coupled with similar results reported in other studies conducted in Tanzania [31, 35].

Other routine and seasonal outdoor activities reported to have possibilities to increase the vulnerability of contracting malaria among school-age children, have been reported by other studies conducted elsewhere as activities exposing all age groups to infections and therefore there is a shared risk of contracting malaria infection to all age groups not specifically to school-age children [30, 32, 34, 36–38].

*Ulalavi* is a most common behaviour among school-age children of 5 to 10 years and could be one of the main possible reasons for higher malaria prevalence among school-age children. The age group is at more risk of mosquito biting when more than three children share a bed, then touching a bed net is inevitable. *Ulalavi* is not a new sleeping behaviour and it has been reported elsewhere as a reason for high malaria prevalence among bed net users, hence affecting the effectiveness of the intervention to protect all age groups against mosquito bites [27], however, results from this study reported ulalavi as common behaviour to younger children than other age groups and that can increase vulnerability to malaria infection among despite the coverage of bed net distribution programmes and the supreme net use reported in the study.

Almost all participants across the study area were unaware of the risk of contracting malaria before bedtime, implying that they could not take any possible protective measures against mosquitoes. Most believe mosquitoes transmit malaria bites at midnight due to knowledge provided by the mass campaigns in Tanzania [39]. Similar perception has been reported by other studies conducted elsewhere [30, 37, 39]. This knowledge gap calls for community education to update and inform the community about the outdoor transmission risk before bedtime and its contribution to residual malaria transmission [30, 31, 34, 37].

Evidence presented in this study should be viewed in line with some limitations. This study focused mainly on activities and behaviour that might increase the vulnerability of school-age children to malaria infection, not what the other groups were doing while the school-age group was engaged in the mentioned activities. However, this does not affect the findings presented. Therefore, there is a need to conduct a mixed-method study to link human and vector species behaviours to explore and quantify risk between age groups at the household level in the study area. A case–control study would be appropriate to quantify and measure infection incidence among school-age children when conducting the mentioned risk behaviours like jando practice, participating in vigodoro, and watching television at kibandaumiza compared to those who are not engaging to the mentioned risk activities and behaviours. if confirmed appropriate measures must be taken to control transmission risk to the age group and the whole community.

## Conclusion

Results of this study indicate potential risk factors for persisting malaria transmission in the three districts of south-eastern Tanzania. The outdoor nighttime activities that keep them in one place for a long period might increase exposure to mosquito bites. Studies to quantify the risk behaviour and activities are recommended to determine magnitude and its implicated impact to the ongoing malaria control interventions. Moreover, results of this study are useful to the NMCP and collaborators in designing interventions that would appropriately target the group to control the parasite reservoir and transmission of the disease [40, 41]. More importantly, it encourages integrating outdoor malaria vector control measures into existing ones to achieve malaria elimination [33]. Also, these findings encourage the improvement of housing [42–46] and call for an urgent need for community education to inform people about outdoor exposure and its contribution to malaria infection, to seal the existing knowledge gap to improve individual malaria protection behaviours against infectious mosquitoes [30, 37, 38, 47, 48].

## Data Availability

Data can be made available upon receipt of official requests, and it must ensure participants’ confidentiality and data privacy.

## Abbreviations

NMCP: National Malaria Control Programme
IHI: Ifakara Health Institution
MUHAS: Muhimbili University of Health and Allied Sciences
1,7-mRCTR: 1,7-Malaria reactive community-based testing and response
ITNs: Insecticide treated nets
IRS: Indoor residual spray
ACOBISREM: Community-Based Integrated Strategies to Reduce Malaria
FGD: Focus Group Discussion
